# Performance, Egg Quality, and Composition in Isa Brown Laying Hens Fed with Different Levels of *Desmodium tortuosum* Leaf Flour

**DOI:** 10.3390/ani14192868

**Published:** 2024-10-05

**Authors:** Gningnini Alain Koné, Tagouèlbè Tiho, N’Goran David Vincent Kouakou, Yapo Magloire Yapi, Konan Raphaël N’Guessan, Margaret Good, Maryline Kouba

**Affiliations:** 1 Unité Mixte de Recherche et d’Innovation Sciences Agronomiques et Procédés de Transformation (UMRI SAPT), Institut National Polytechnique Felix Houphouët Boigny, Yamoussoukro BP 1313, Côte d’Ivoire; tagouelbe.tiho@inphb.ci (T.T.); david.kouakou@inphb.ci (N.D.V.K.); magloire.yapi@inphb.ci (Y.M.Y.); raphael.nguessan@inphb.ci (K.R.N.); 2 Independant Researcher and Private Consultant, A96DX4C Dun Laoghaire, Ireland; mgood94@yahoo.co.uk; 3 Unité Mixte de Recherche Physiologie, Environnement et Génétique pour l’Animal et les Systèmes d’Élevage (UMR PEGASE), Institut Agro, National Research Institute for Agriculture, Food and Environment (INRAE), 35590 Saint Gilles, France; maryline.kouba@agrocampus-ouest.fr

**Keywords:** African plant, egg color, egg cholesterol, egg composition, layer, Cote D’Ivoire

## Abstract

**Simple Summary:**

There is a high demand for eggs in developing countries. Poultry feed is expensive; thus, farmers cannot afford to buy commercial feed. Therefore, it is important to explore the qualities of potential new feed materials for egg production. *Desmodium tortuosum* is a common weed in sub-Saharan Africa. These feed materials can be used for supplementing laying hen diets. Three hundred hens were used for 13 weeks. Two control groups received diet W, based on white corn, or diet Y containing yellow corn. The other groups received a diet based on white corn supplemented with *Desmodium tortuosum* leaf flour at 2.5%, 5%, 7.5%, or 10%. Diets supplemented with *Desmodium* improved laying performance and yolk color at a reduced feed cost per egg but had no effect on cholesterol content. The inclusion of *Desmodium tortuosum* leaf flour in a white corn-based diet for laying hens is effective in lowering feed cost, increasing egg production, and improving yolk color.

**Abstract:**

The objective of this study was to investigate the effects on laying performance, egg quality traits, color, and composition of supplementing a white corn-based diet with *Desmodium tortuosum* leaf flour. Three hundred 32-week-old hens were distributed to 30 pens of 10 hens each and allocated to six dietary treatments (five replicates per treatment) for 13 weeks. Two control groups of 50 hens received one of either diet Y, based on yellow corn, or diet W, based on white corn. The other groups received a diet based on white corn supplemented with *Desmodium tortuosum* leaf flour at 2.5%, 5%, 7.5%, or 10% (diet D). Diet D improved laying performance and yolk color at a reduced feed cost per egg but had no effect on cholesterol content. In conclusion, the inclusion of *Desmodium tortuosum* leaf flour, in a white corn-based diet, is effective in lowering feed cost, increasing egg production, and improving yolk color.

## 1. Introduction

Commercial poultry farming has grown strongly in many African countries over the past ten years. This increase is due to a high demand for eggs and poultry meat and consequently, the investment in poultry projects [1]. Over the same period, there has been a rapid development in the population and living standards, leading to a high demand for poultry. However, rapidly expanding demands for poultry products are not really stimulating the local poultry production as much as might be expected because of high levels of poultry product imports [2].

The egg consumption rate is 64 eggs per Ivorian per year [3] and projections indicate a consumption between 100 and 150 eggs per Ivorian per year by 2030 [4]. Local poultry production in Africa has been neglected for a long time because of its low productivity, even if the taste of the meat and eggs from these animals is highly appreciated by the population. This preference for local products has been shown in several studies, for eggs in South Africa [5] and Ethiopia [6] and poultry meat in Ghana [7].

The cost of feed is limiting the development of both commercial and local breeds of poultry [2]. Feed represents more than 60% of the total cost of egg production [8]. Sourcing a cheap, safe, and available feed is a huge priority for the developing world [2]. Indeed, the limited supply of cheap quality feed seems to be preventing an increase in poultry production [2].

Many studies have been undertaken to evaluate feed resources for livestock nutrition to minimize production costs without altering performance [9]. The authors are unaware of any such studies, comparing the use of white corn instead of yellow corn in laying hen nutrition. Yellow corn (*Zea mays*) is very frequently used as a dietary component for laying hens. In Côte d’Ivoire, white corn is more readily available and less expensive for livestock nutrition than yellow corn (0.33 vs. 0.42 €/kg feed, respectively). The problem when using white corn in laying hens is its low pigment content, such as carotenoids, necessary for yolk coloration.

Indeed, yolk coloration is desirable and actually needed because it is a consumer preference, and consumers think that deep-yellow-color yolks indicate free range and better conditions for the laying hens [10]. Thus, this trial also incorporated a study comparing diets based on yellow corn with that based on white corn and also the effects of supplementing a white corn-based diet for laying hens with a local plant rich in carotenoids.

*Desmodium tortuosum* is an abundant indigenous leguminous plant in Africa considered as one of the most troublesome weeds in peanut crops. It is therefore freely available and is usually used as a fodder for rabbits [11].

In the present study, we compared diets based on yellow corn and white corn and also tested several levels of supplementation of *Desmodium tortuosum* leaf flour in white corn-based diets for local Isa Brown laying hens.

## 2. Materials and Methods

### 2.1. Ethics Statement

The animals used were reared in compliance with regulations for the humane care and use of animals in research, according to the EU directive 86/609. National Authorization to Experiment on live animals n°3502 has been delivered to M. Kouba by the French Minister of Agriculture. In this study, there was no need for ethical approval due to the lack of blood sampling from the animals and the absence of any surgical procedures. The animals were raised according to the European Council Directive 1999/74/EC and were only weighed in the study.

### 2.2. Animals and Experimental Design

The study was conducted from May to July 2024 at the experimental farm of the National Polytechnic Institute Felix Houphouët Boigny (INP-HB), Yamoussoukro, Côte d’Ivoire, in collaboration with the French National Research Institute for Agriculture, Food and Environment (INRAE).

*Desmodium tortuosum* was collected locally from around the INP-HB. *Desmodium tortuosum* leaf flour was prepared by grinding leaves after they were oven-dried (60 °C) over three full days. All the diets for the study were formulated at the Animal Science Laboratory of INP-HB to meet laying hen physiological requirements and to be isoproteic and isoenergetic. The composition of *Desmodium tortuosum* leaf flour was reported earlier [12] and is provided in this manuscript in Table 1.

A total of 300 Isa Brown laying hens, at the peak stage of egg production (32 weeks old) with an average weight of 1569 g, were randomly distributed in 50 pens of ten hens each and allocated to ten dietary treatments (five replicates (pens) per treatment). Two control groups of hens (50 birds in each) received a control diet; one group received diet Y based on yellow corn, and the other group diet W, based on white corn. The trial groups received a diet based on white corn supplemented with *Desmodium tortuosum* leaf flour at 2.5% (D2.5), 5% (D5), 7.5% (D7.5), or 10% (D10), as shown in Table 2, which presents the ingredients and composition of the six diets for laying hens.

Each pen was 4 m^2^ (2 m length, 2 m width), representing 1000 cm^2^ per laying hen. All pens were located in a covered well-ventilated building and each pen contained a nest, perches, a feeder, a drinking trough, and fresh litter. These rearing conditions fulfilled laying hen requirements [13]. The lighting program was (16L:8D). Water was available ad libitum and feed was distributed twice daily (at 9 o’clock am and 5 o’clock pm) with a fixed quantity (150 g feed/day/laying hen) to avoid the waste of feed, frequently observed in local breeds of hens. The health and mortality of hens were monitored daily. The total duration of the trial was 91 d (7 d of adaptation to the environmental conditions to be assured that all hens were healthy and in active production, and 84 d on trial, meaning that the trial started with 33-week-old laying hens).

### 2.3. Egg Physical Parameters

At d28, d56, and d84 of the trial, five eggs per pen were collected to determine the egg weight, vitellus weight, albumen weight, and shell weight. The shell thickness of eggs was determined (with the shell membrane) using the mean value of measurements from three locations (air cell, equator, and sharp end) of the egg, using a micrometer (General Tools & Instruments, New York, NY, USA) with a 0.01 mm accuracy. Haugh units were measured with an Haugh tester, Bröring Egg Quality 3.0 (BRÖRING Technology GmbH, Saint-Laurent-du-Var, France).

Yolk color was determined using the DSM Yolkfan™ lineal (DSM Nutritional Products France, La Garenne-Colombes, France) and colors were scored according to 15 sample colors ranging from 1 (the lightest) to 15 (the darkest). The egg yolk color was also analyzed with Minolta Chroma Meter CM-1000R (Konika Minolta, Ramsey, NJ, USA) using the CIE (Commission Internationale d’Eclairage) Lab scale. The L*, a*, and b* values reflect brightness, redness, and yellowness, respectively. The Haugh unit (HU) which denotes egg freshness was determined by the formula HU = 100 × log (h − 1.7 × W^0.37^ + 7.6), where h = height of the thick albumen at 1 cm from the yolk and W = egg weight [3].

### 2.4. Chemical Analyses

Total fiber content was determined for each of the ten diets using a Fibretec System 1021 Cold extractor (Saint André de Cubzac, France) (Table 2). Samples of diets and selected eggs were analyzed for dry matter, ash, crude protein (N 6.25), and carotenoid content in accordance with AOAC [14]. In summary, water content was determined according to AOAC 950.46; samples were dried in the oven at 105 °C; ash content was determined by mineralization of samples at 550 °C according to AOAC 920.153; total protein (crude protein, N 6.25) content was assessed by the Kjeldahl method according to AOAC 928.08; and carotenoid analysis was performed according to the spectrophotometric AOAC official method 970-64 (at λ = 450 nm). Lipids were extracted from the diet samples and the selected eggs by the chloroform/methanol procedure [15]. The fatty acid (FA) profile was analyzed for *Desmodium tortuosum* leaf flour. Fatty acid composition was measured after the methylation of samples. Fatty acid methyl esters were prepared with brome trifluoride methanol according to [16] and analyzed on an Agilent Technologies 6890 N Gaz chromatograph (Bios Analytic, Toulouse, France) with an internal standard (C21:0, Sigma-Aldrich, Saint-Quentin-Fallavier, France) used to quantify FAs (g/100 g of total FAs). Total egg cholesterol content was quantified using a commercial kit (Giesse Diagnostics SRL, Guidonia Montecelio, Italy) according to a colorimetry method [17].

### 2.5. Statistical Analysis

Data were analyzed by one-way analysis of variance ANOVA, an option of the generalized linear model (GLM) of R version 4.4.0 software (Copyright © 2024, R Foundation for Statistical Computing Platform: x86_64-apple-darwin20) with diet as the main effect. The statistical model used was Y_iik_ = μ + D_i_ + R_ij_ + γijk, where Y_ijk_ = response variables from each individual replication; μ = the overall mean; D_i_ = the effect of diet; R_ij_ = the inter-trial unit (replications) error term; and γijk = the intra-trial unit error term. Least significant difference comparisons were made between treatment means per diet for main effects where there was a significant F value. Significance implies *p* < 0.05 unless stated otherwise.

## 3. Results

### 3.1. Diet Composition

The composition of *Desmodium tortuosum* leaf flour is presented in Table 1 and the characteristics of the six diets are presented in Table 2. The six diets are isonitrogenous and isocaloric. The proportion of dry matter was higher for the four diets supplemented with Desmodium (D2.5, D5, D7.5, and D10), whatever the level of inclusion. The β-carotene content in laying hens increased with the level of supplementation with Desmodium, whatever the level of supplementation. Proportions of ashes and lipids were similar in all the diets.

### 3.2. Laying Hen Performance

The effect of the different control diets (Y and W) and diets supplemented with Desmodium (diet D) on the performance of laying hens is presented in Table 3. There was no mortality during the trial. Feeding diet D led to the highest egg production and the lowest feed-to-egg ratio compared with the other diets (*p* = 3.24 × 10^−16^ and *p* = 6.62 × 10^−16^, respectively). There was an increase in egg production with increased levels of Desmodium supplementation (3.24 × 10^−16^). The egg production from hens fed diet D was highly correlated with the level of supplementation (r^2^ = 0.98). Diet D significantly improved the egg production by 10.00% compared with the control diet W and by 12.10% compared with diet Y (*p* = 3.24 × 10^−16^). The level of Desmodium supplementation had no effect on the laying hen final body weight (*p* = 3.57 × 10^−1^).

### 3.3. Egg Quality Traits

As shown in Table 4, there was no diet effect on the albumen percentage and Haugh units (*p* = 1.72 × 10^−1^). There was also no diet effect on egg weight, except for the group fed diet Y, in which egg weight was the lowest compared with the other diets (*p* = 8.78 × 10^−3^). The shell percentage and eggshell thickness of eggs from hens fed diet D was similar to the eggs from hens fed the control diets W or Y. The feed cost was on average lower with diets supplemented with Desmodium than with diets W and Y (mean of 5.04 vs. 5.71 and 6.59 euro cents per egg, respectively).

### 3.4. Egg Color

Color and β-carotene content of yolks from laying hens fed the six diets are presented in Table 5. Diet W gave the lowest color value with the DSM Yolkfan compared to the other diets, whereas diet D gave the darkest yellow color (*p* = 1.20 × 10^−16^). The value of the color of yolks and their β-carotene content in laying hens increased with the level of supplementation with Desmodium (*p* = 2.13 × 10^−12^). The β-carotene content of egg yolk from laying hens fed diets D7.5 and D10 was higher than the content of the other egg yolks from laying hens fed diet Y. The parameters L*, a*, and b* showed a similar evolution than the yolk color estimated with the colorimetric fan. Values of lightness L* were the lowest for eggs of laying hens fed diet D; the lightness value was the highest with diet W compared to the other diets (*p* = 2.20 × 10^−15^). The chrome a* value was the highest with diets D and Y compared to diet W (*p* = 1.33 × 10^−13^). The chrome b* value was the highest with diets D7.5, D10, and Y, followed by diets D5 and D2.5; the lowest value was obtained with diet W (*p* = 2.61 × 10^−15^).

### 3.5. Egg Chemical Composition

As shown in Table 6, there is no desmodium-supplemented diet effect on albumen dry matter, ash, and protein percentage (*p* = 1.96 × 10^−1^*, p* = 1.28 × 10^−1^ and *p* = 7.90 × 10^−1^, respectively). Some vitellus components (dry matter, ash, protein, and lipids) were not affected by the diet supplemented with desmodium (*p* ≥ 0.05), whereas egg cholesterol content was affected by the diets (*p* = 1.15 × 10^−12^). The lowest value of egg cholesterol content (9.51 mg/g of egg) was obtained with diet D10.

## 4. Discussion

To our knowledge, the inclusion of *Desmodium tortuosum* in white corn-based diets of laying hens has not been previously reported. In our study, there was no mortality during the trial. The lack of effect of the diet on laying hen weight has already been demonstrated [8,18].

In this study, we used ISA Brown laying hens and found that the egg production was similar to the performance of 34-week-old Isa Brown hens in Bangladesh [19], similar to the results of [20,21] in 43- and 44-weeks-old Isa Brown hens in Korea, respectively. The beneficial effects of dietary supplementation with plant extracts on intestinal health, intestinal integrity, and nutrient utilization have been reported [22,23], which can improve laying hen performance. In the present study, egg production was increased by the supplementation of the diets compared to control diets (0.89 egg produced/hen, 0.85 egg produced/hen, 0.82 egg produced/hen, and 0.80 egg produced/hen with diets D, W, and Y, respectively). These results agree with [22], which reported that feeding peppermint to laying hens had a positive influence on the conversion of digested diet into eggs. Moreover, the antimicrobial and antioxidant properties of bioactive components in both herbal plants have been reported [24,25], and they play a crucial role in the digestion and absorption of nutrients [26] that might have improved the performance parameters of laying hens [21,27,28]. On the other hand, the better feed-to-egg ratio observed due to the inclusion of plant *Desmodium tortuosum* could be due to the increased diet utilization efficiency [22].

We found the same results for guinea fowl diets supplemented with Hevea seed meal [3]. A higher egg production as compared to controls has also been observed previously with diets supplemented with Hevea seed oil [29,30] and with diets supplemented with fermented Hevea leaves and seeds [31].

However, there was an increase in egg production when levels of supplementation with Desmodium leaf flour increased, which is contrary to the results presented by [8], which used diets supplemented with *Desmodium uncinatum* and found a decrease in egg production with the increase in the level of the plant in the diet. However, the plant used in their study, *Desmodium uncinatum*, is not similar to the plant *Desmodium tortuosum* used in the present study.

Egg weight was similar with diets W and D and lower with diet Y, which is the diet traditionally used in Cote d’Ivoire. The egg weight in the present study was in the same range than results obtained in [19] but was lower than eggs of 46-week-old Isa Brown hens in Korea [20] or eggs of 45-week-old Isa Brown hens in Thailand [32]. Compared to Isa Brown hens of 46 weeks of age [33], the percentage of shell was similar, but eggs of the present study exhibited a higher yolk proportion and a similar albumen percentage. The results of the present study comply with the percentages provided by [34], whose author said in his review that an egg consists of a shell at 9–12%, albumen at 60%, and yolk at 30–32%.

Shell thickness was similar to Isa Brown hen’s eggs in the studies of [20,21] but lower than the results of [32,35]. In the present study, diet did affect eggshell thickness, which was not an effect observed in guinea fowl fed Hevea seed meal [3] or in laying hens fed a diet supplemented with Hevea fermented leaves and seeds [31]. A 2015 review concluded that if diet affects eggshell quality, the change is often of low magnitude, especially in young layers [36].

The Haugh unit is an important item in evaluating albumen quality and egg freshness. The Haugh unit, which indicates the relationship of the height of the thick white to the weight of the egg, is the most widely used measure of the albumen quality and freshness of eggs [37]. As albumen quality decreases, Haugh units also decrease. In the present study, the value of the Haugh unit was very high, from 91 to 98.35, demonstrating the quality of the eggs produced by layers, and there was no observed dietary effect. There was also no diet effect on the Haugh unit in the study on guinea fowl where the values were lower than those observed in the present trial [3]. The Haugh unit values were in the range of the Isa Brown’s egg in the study of [35] in Nigeria, but higher than those for Isa Brown hens’ eggs in the studies of [20,21,32,33].

Yolk color is an important esthetic factor, which determines product purchase [10] and an essential feature of egg quality [28], where African consumers prefer a deep yellow to a pale-yellow coloration [3,5,38]. The carotenoid content of the eggs is not only an esthetic value, but it has several other positive roles (antioxidant, provitamin) [39,40]. The color value with the DSM Yolkfan of the eggs produced in this study varied from pale yellow to deep yellow, (8.83 to 11.33 with diet D). The concentration of carotenoid pigments determines the intensity of the egg yolk color. This is the reason why the present study showed an increase in the color value with the increase in the level of supplementation with *Desmodium tortuosum* leaf flour in the diets. The *Desmodium tortuosum* leaf flour contains a considerable amount of carotenoids [41], which may be responsible for better yolk color in the Desmodium-fed groups. However, we did not analyze the total carotenoid content of *Desmodium tortuosum* leaf flour in this study to confirm this assumption. But a high correlation (r^2^ = 0.89) between the β-carotene content of diet D and the level of supplementation could confirm this assumption.

This result was also observed using diets with Hevea seed oil supplementation from 1 to 6% [42]. The β-carotene content of egg yolks from hens fed diet D was even higher than the β-carotene content of egg yolks from hens fed diet Y (the routine diet used in Côte d’Ivoire). The β-carotene content of egg yolks from hens fed diet D was highly correlated with the color of the eggs, whatever the level of supplementation (r^2^ = 0.99).

An albumen protein content in the range of the results of the present study was found for Lohmann Brown laying hens, but with a higher vitellus protein content [43].

The egg cholesterol content found in this study was in the same range as in some studies using Isa Brown laying hens [21,32] but was lower than the results of [19,33]. Yolk cholesterol content decreased in D10 groups compared with the other groups. Egg cholesterol has always been a concern in relation to human health. A recent review concluded that a high daily egg consumption may increase cardiovascular disease risks [44]. For sub-Saharan Africans, egg consumption is linked to an increase in plasma cholesterol content and incidents of cardiovascular diseases, and this certainty has led to a reduction in egg intake [37]. Omega 3 fatty acids represent 36.50% of the content in Desmodium leaf flour [12]. This high amount of omega 3 fatty acids explains the depressive effect of Desmodium leaf flour on cholesterol concentration also observed in guinea fowl [3,45]. It also explains the decrease in cholesterol contents. A decrease in yolk cholesterol content as compared to the control group was also observed in groups of hens receiving Hevea seed oil [29]. Moreover, the high level of fiber in diet D10 could explain the depressive effect of Desmodium leaf flour on cholesterol concentration. In fact, it is very well known that dietary fiber increases the removal of cholesterol [28,46,47].

The use of a feed supplemented with dried orange pulp or dried citrus pulp up to, respectively, 10% and 12% in laying hens for, respectively, 8 and 12 weeks did not affect liver function based on serum AST levels [28,48]. There was no mortality during the trial. This result suggested that the hepatic function was not adversely affected by the dietary inclusion of *Desmodium Tortuosum* leaf flour at up to 10% in laying hens. Aspartate aminotransferase (AST) of blood is a useful marker of hepatic function and health [28]. However, we did not analyze the AST level of blood in this study to confirm this assumption. The diets supplemented with humic plant preparations to a limited degree in Lohmann Brown hens for 44 weeks affected the values of blood alanine aminotransferase (ALT), aspartate aminotransferase (AST), and alkaline phosphatase (ALP) [49]. It is difficult to determine, based on this study, the effects of the long-term use of high levels of *Desmodium Tortuosum* leaf flour on the health of laying hens.

## 5. Conclusions

It is possible to use *Desmodium tortuosum* leaf flour with a diet based on white corn in laying hens at a very low cost. Egg yolks from hens fed diets that were supplemented with *Desmodium tortuosum* leaf flour had the highest carotenoid content. The use of such a diet increased the performance of the bird and improved the egg yolk color compared with the control diet Y containing yellow corn. This improvement in yolk color by the diet containing *Desmodium tortuosum* leaf flour was due to its high content of carotenoids.

## Figures and Tables

**Table 1:** Composition of *Desmodium tortuosum* used in laying hen diets.

Item	*Desmodium tortuosum* Leaf Flour
Dry matter (% FM)	22.90
Ashes (% DM)	10.70
Crude fiber (% DM)	24.90
Crude protein (% DM)	19.50
Lipids (% DM)	2.40
Fatty acid profile (in % of total fatty acids)	
∑SFA	37.80
∑MUFA	6.50
∑PUFA	55.70
∑n-6 PUFA	19.20
∑n-3 PUFA	36.50
∑n-6 PUFA/∑n-3 PUFA	0.50

Abbreviations: (FM) fresh matter; (DM) dry matter; (SFA) saturated fatty acid; (MUFA) monounsaturated fatty acid; (PUFA) polyunsaturated fatty acid. Adapted from [12].

**Table 2:** Ingredients and composition of controls and trial diets.

Item	Diets					
	Controls		Desmodium Supplemental Levels (%)			
	Y	W	D2.5	D5	D7.5	D10
Yellow corn	565.00	0.00	0.00	0.00	0.00	0.00
White corn	0.00	565.00	550.80	536.70	522.60	508.20
Soybean meal	130.00	130.00	126.80	123.40	120.30	117.00
Wheat bran	100.00	100.00	97.50	95.00	92.50	90.00
Fishmeal	105.00	105.00	102.40	99.80	97.10	94.50
Shellfish	85.00	85.00	82.90	80.80	78.60	76.80
*Desmodium tortuosum*	0.00	0.00	25.00	50.00	75.00	100.00
Vitamin–mineral premix ^1^	15.00	15.00	14.60	14.30	13.90	13.50
Analyzed composition (%) ^2^						
Dry matter	88.00	87.90	88.00	88.00	88.10	88.10
Ashes	10.10	10.10	10.10	10.20	10.20	10.20
Protein	20.80	21.00	21.00	21.00	20.90	20.90
Lipid	3.60	3.40	3.40	3.40	3.30	3.30
Crude fiber	8.10	7.80	8.20	8.70	9.10	9.50
β-carotene (mg/kg)	1.08	0.31	0.98	1.11	1.34	1.51
Calculated composition (g/kg)						
Lysine	8.90	9.00	9.00	8.90	8.90	8.80
Methionine	3.10	3.40	3.40	3.40	3.40	3.30
Calcium	36.00	35.30	34.80	35.60	35.80	35.70
Available phosphorus	6.10	5.80	5.70	5.60	5.50	5.40
ME (kcal/kg)	2721.01	2704.03	2704.04	2704.02	2705.01	2705.02
Price (€/kg)	0.44	0.39	0.38	0.38	0.38	0.37

Abbreviations: (Y) control diet based on yellow corn; (W) control diet based on white corn; (D2.5) W plus 2.5% *Desmodium tortuosum* leaf flour; (D5) W plus 5% *Desmodium tortuosum* leaf flour; (D7.5) W plus 7.5% *Desmodium tortuosum* leaf flour; (D10) W plus 10% *Desmodium tortuosum* leaf flour. ^1^ Vitamin–mineral premix supplied the following per kilogram of diet: vitamin A (retinyl acetate), 3.75 mg; vitamin D3 (cholecalciferol), 62.50 µg; vitamin E (α-tocopherol), 30.15 mg; vitamin K3 (menadione), 4 mg; vitamin B1 (thiamine), 2 mg; vitamin B2 (riboflavin), 8 mg; vitamin B6 (pyridoxine-HCl), 4 mg; vitamin B12 (cyanocolabamine), 0.04 mg; biotin, 0.12 mg; folic acid, 2 mg; niacin, 37 mg; Co, 1.2 mg; Cu, 8 mg; Fe, 60 mg; I, 1 mg; Mn, 74 mg; Se, 0.6 mg; Zn, 75 mg; endo-1.4-beta-xylanase; 100 U/kg; 6 phytase, 1500 U/kg. ^2^ Values are the means of three analyses per sample.

**Table 3:** Effect of control diets and level of *Desmodiun tortuosum* supplementation on performance in laying hens.

Item	Diets						SEM	*p*-Value
	Controls		Desmodium Levels (%)					
	Y	W	D2.5	D5	D7.5	D10		
IBW (kg)	1.58	1.56	1.59	1.58	1.56	1.62	0.16	9.99 × 10^−1^
FBW (kg)	2.11	2.22	2.04	2.06	2.45	2.05	0.22	3.57 × 10^−1^
EP (%)	80.01 ^d^	82.12 ^cd^	84.32 ^c^	90.40 ^b^	92.51 ^ab^	96.60 ^a^	0.94	3.24 × 10^−16^
DFI (g)	119.80	120.01	119.80	119.30	119.02	118.40	1.48	9.50 × 10^−1^
FER	2.83 ^a^	2.53 ^b^	2.52 ^b^	2.40 ^c^	2.34 ^cd^	2.16 ^d^	0.05	6.62 × 10^−16^

Abbreviations: (Y) control diet based on yellow corn; (W) control diet based on white corn; (D2.5) W plus 2.5% *Desmodium tortuosum* leaf flour; (D5) W plus 5% *Desmodium tortuosum* leaf flour; (D7.5) W plus 7.5% *Desmodium tortuosum* leaf flour; (D10) W plus 10% *Desmodium tortuosum* leaf flour; (IBW) initial body weight; (FBW) final body weight; (EP) egg production; (DFI) daily feed intake; (FER) feed-to-egg ratio; (SEM) standard error of the mean. ^a,b,c,d^ Parameter means within rows of diet with no common superscript differ (*p* < 0.05). Each value represents the mean of 50 laying hens per diet.

**Table 4:** Effect of control diets and level of inclusion of *Desmodiun tortuosum* on egg physical parameters and egg feed cost.

Item	Diets						SEM	*p*-Value
	Controls		Desmodium Levels (%)					
	Y	W	D2.5	D5	D7.5	D10		
EFC	6.59	5.71	5.42	5.04	4.86	4.52		
EW (g)	52.84 ^b^	57.94 ^a^	56.61 ^a^	55.18 ^a^	54.77 ^a^	56.41 ^a^	1.10	8.78 × 10^−3^
Sh (%)	12.54	12.39	13.04	12.56	12.44	12.80	0.38	1.24 × 10^−1^
Vit (%)	31.33	30.74	32.25	31.40	29.24	29.64	0.96	1.47 × 10^−1^
Alb (%)	56.13	56.83	54.71	56.04	58.32	57.56	1.09	4.82 × 10^−1^
ShT (mm)	0.39	0.42	0.41	0.36	0.36	0.36	0.02	1.17 × 10^−1^
HU	94.11	92.87	95.88	94.71	94.70	98.35	2.10	1.72 × 10^−1^

Abbreviations: (Y) control diet based on yellow corn; (W) control diet based on white corn; (D2.5) W plus 2.5% *Desmodium tortuosum* leaf flour; (D5) W plus 5% *Desmodium tortuosum* leaf flour; (D7.5) W plus 7.5% *Desmodium tortuosum* leaf flour; (D10) W plus 10% *Desmodium tortuosum* leaf flour; (EFC) egg feed cost in euro cents; (EW) egg weight; (Sh) shell; (Vit) vitellus; (Alb) albumen; (ShT) shell thickness; (HU) Haugh unit; (SEM) standard error of the mean. ^a,b^ Parameter means within rows of diet with no common superscript differ (*p* < 0.05). Each value represents the mean of 75 eggs per diet (15 eggs per replicate).

**Table 5:** Effect of control diets and level of inclusion of *Desmodiun tortuosum* on the CIELAB L*, a*, and b* color space and YolkFan’s rating of yolk.

Item	Diets						SEM	*p*-Value
	Controls		Desmodium levels (%)					
	Y	W	D2.5	D5	D7.5	D10		
L*	78.42 ^c^	82.01 ^a^	80.19 ^b^	78.78 ^c^	77.43 ^cd^	75.89 ^d^	0.10	2.20 × 10^−15^
a*	6.63 ^c^	1.64 ^e^	4.03 ^d^	6.17 ^c^	8.36 ^b^	10.63 ^a^	0.29	1.33 × 10^−13^
b*	146.46 ^a^	71.44 ^d^	111.63 ^c^	133.22 ^b^	152.02 ^a^	149.08 ^a^	1.79	2.61 × 10^−15^
Yolk color ^1^	9.83 ^b^	4.17 ^d^	8.83 ^c^	10.08 ^b^	11.00 ^a^	11.33 ^a^	0.10	1.20 × 10^−16^
β-carotene (mg/kg)	1.75 ^b^	0.63 ^d^	1.56 ^c^	1.82 ^b^	1.92 ^a^	1.95 ^a^	0.08	2.13 × 10^−12^

Abbreviations: (Y) control diet based on yellow corn; (W) control diet based on white corn; (D2.5) W plus 2.5% *Desmodium tortuosum* leaf flour; (D5) W plus 5% *Desmodium tortuosum* leaf flour; (D7.5) W plus 7.5% *Desmodium tortuosum* leaf flour; (D10) W plus 10% *Desmodium tortuosum* leaf flour; (SEM) standard error of the mean. ^1^ According to a scale ranging from 1 (the lightest) to 15 (the darkest). ^a,b,c,d,e^ Parameter means within rows of diet with no common superscript differ (*p* < 0.05). Each value represents the mean of 75 eggs per diet (15 eggs per replicate).

**Table 6:** Effect of control diets and level of inclusion of *Desmodiun tortuosum* on egg composition.

Item	Diets						SEM	*p*-Value
	Controls		Desmodium Levels (%)					
	Y	W	D2.5	D5	D7.5	D10		
Albumen composition (%*w*/*w*)								
Dry matter	14.86	13.67	14.01	13.99	13.56	13.72	0.56	1.96 × 10^−1^
Ash	0.88	0.82	0.83	0.77	0.84	0.79	0.07	1.28 × 10^−1^
Protein	13.36	11.44	12.88	12.87	12.39	12.57	0.49	7.90 × 10^−1^
Vitellus composition (%*w*/*w*)								
Dry matter	46.47	48.34	49.06	48.31	49.97	47.15	2.60	1.43 × 10^−1^
Ash	2.37	2.32	2.36	2.70	2.53	2.67	1.14	1.39 × 10^−1^
Protein	13.74	11.66	11.87	13.16	14.67	13.11	1.45	3.48 × 10^−1^
Lipids	30.36	34.35	34.82	32.45	32.77	31.37	1.61	3.31 × 10^−1^
Cholesterol (mg/g of egg)	9.95 ^a^	10.05 ^a^	10.13 ^a^	10.23 ^a^	9.88 ^a^	9.51 ^b^	0.44	1.15 × 10^−12^

Abbreviations: (Y) control diet based on yellow corn; (W) control diet based on white corn; (D2.5) W plus 2.5% *Desmodium tortuosum* leaf flour; (D5) W plus 5% *Desmodium tortuosum* leaf flour; (D7.5) W plus 7.5% *Desmodium tortuosum* leaf flour; (D10) W plus 10% *Desmodium tortuosum* leaf flour; (SEM) standard error of the mean. ^a,b^ Parameter means within rows of diet with no common superscript differ (*p* < 0.05). Each value represents the mean of 75 eggs per diet (15 eggs per replicate).

## Data Availability

The data presented in this study are available in the study.
